# Generative Adversarial Networks to Improve Fetal Brain Fine-Grained Plane Classification

**DOI:** 10.3390/s21237975

**Published:** 2021-11-29

**Authors:** Alberto Montero, Elisenda Bonet-Carne, Xavier Paolo Burgos-Artizzu

**Affiliations:** 1 Faculty of Computer Science, Multimedia and Telecommunications, Universitat Oberta de Catalunya, 08018 Barcelona, Spain; alberto.montero.agudo@gmail.com (A.M.); ebonetca@uoc.edu (E.B.-C.); 2 BCNatal, Barcelona Center for Maternal-Fetal and Neonatal Medicine (Hospital Clinic and Sant Joan de Deu), 08028 Barcelona, Spain; 3 Escola Tècnica Superior d’Enginyeria de Telecomunicació de Barcelona (ETSETB), Universitat Politecnica de Catalunya-BarcelonaTech, 08034 Barcelona, Spain

**Keywords:** generative adversarial networks, deep learning, ultrasound image classification

## Abstract

Generative adversarial networks (GANs) have been recently applied to medical imaging on different modalities (MRI, CT, X-ray, etc). However there are not many applications on ultrasound modality as a data augmentation technique applied to downstream classification tasks. This study aims to explore and evaluate the generation of synthetic ultrasound fetal brain images via GANs and apply them to improve fetal brain ultrasound plane classification. State of the art GANs *stylegan2-ada* were applied to fetal brain image generation and GAN-based data augmentation classifiers were compared with baseline classifiers. Our experimental results show that using data generated by both GANs and classical augmentation strategies allows for increasing the accuracy and area under the curve score.

## 1. Introduction

Diagnostic ultrasound is an essential tool during pregnancy [1]. It is employed both as a screening tool as well as to better assess high risk patients, both during early [2] or late pregnancy [3]. Examples are the measurement of fetal biometries to monitor fetal growth and weight [4], Doppler blood flow to study blood circulation [5] or nuchal translucency measurement, which is the basis for the first trimester screening of fetal aneuploidies [6].

The acquisition of fetal and maternal ultrasound images is done following international guidelines promoted by scientific committees [7]. These guidelines provide clear protocols on which images need to be acquired depending on the trimester of pregnancy and classification of the patient. This results in each ultrasound examination having a large number of images (typically, more than 20). Three dimensional (3D) images and videos can also be acquired to complete the clinical examination.

Then, as a first step in any protocol both in the clinical and research settings, the images acquired during the examination have to be classified. Having a clinician or trained technician manually select and classify the images is slow and prone to mistakes. Being able to automatically classify the images acquired during an ultrasound examination can prove very useful to increase cost-effectiveness and reduce human errors in the process [8].

Prior works addressing this problem have relied on gathering a large amount of manually labeled data and then apply deep supervised learning methods [8,9,10,11,12]. General fetal planes can be now reliably detected from the latest ultrasound machines [12] thanks to these prior efforts and public data is now available [13].

**Figure 1:**
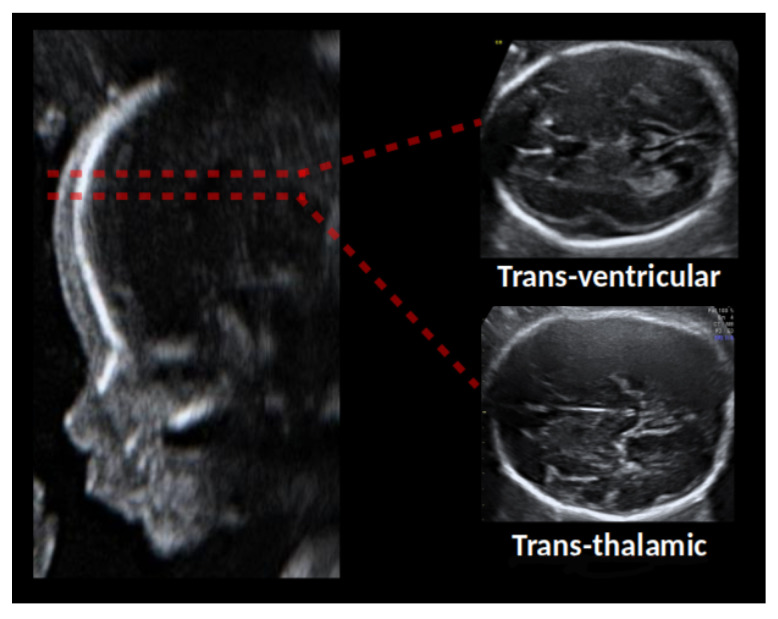
Fetal brain plane images used in this study [8].

However, classifying images with fetal anomalies or distinguishing between the different axial brain planes (see Figure 1) where images are very similar to each other and hard to classify even for domain experts, is still an unsolved problem. And for these cases, labeled data is not readily available to the majority of medical or research centers. One of the possible solutions is to use generative models such as Generative adversarial networks (GANs) [14] to generate artificial images that can then be used to augment the number of examples available to train the classifiers.

In this study, our main objective is to assess if the latest state-of-the-art GANs can help deep learning ultrasound classifiers. We focus on ultrasound fetal brain fine-grained classification (distinguishing trans-thalamic from trans-ventricular axial plane images), see Figure 1. This is a hard classification task and there is not a large number of images readily available, making it ideally suited for the purpose of this study.

The main contributions of this study are two:

1. Evaluate state-of-the-art GANs such as StyleGAN family of architectures [15,16] on fetal ultrasound images. These models are capable of generating highly realistic high resolution images of human faces and other objects, but as far as we know, this is the first time StyleGAN2 networks are used as data augmentation method for ultrasound image classification.
2. Evaluate if the artificial images generated by these models can benefit deep learning supervised classifiers. We evaluated two scenarios (1) improving their accuracy by augmenting total number of training images (augmentation experiments, see Section 4.3.1) and (2) testing if similar accuracy can be achieved with fewer real examples (replacement experiments, see Section 4.3.2).

## 2. Related Work

Generative adversarial networks [14] have obtained a lot of attention due to the realistically looking artificial images they are capable of generating. First GAN model [14] was formed by multilayer perceptrons, while the first deep convolutional GAN (DCGAN) [17] instead exploited the successful application of convolutional neural networks to GANs. Both GANs were defined under a unsupervised or unconditional framework, where image generation is performed from random noise without additional information from classes or any kind of conditional information.

In medical imaging, first DCGANs and variants were mainly applied to generate realistically looking low resolution artificial images, with resolutions ranging from 16 × 16 to 64 × 64. Examples are the generation of 16 × 16 prostate lesions [18] or 56 × 56 lung cancer nodules [19]. Later, GANs have been shown to be useful in many medical imaging applications such as image reconstruction, segmentation, detection, classification, and cross-modality synthesis to overcome issues related to scarcity and class imbalance [20].

Beyond 128 × 128 resolution, good quality artificial images are difficult to obtain with classical DCGANs. Methods to progressively grow GANs [21] have been applied in [22] on 256 × 256 skin lesion images, or in [23] where 1280 × 1024 images of mammograms where generated. Still, ref. [24] showed that when dealing with limited and high variance data, DCGANs performance decreases notably from 64 × 64 to 128 × 128 up to 256 × 256 resolution images. Most works based on DCGANs and variants have been successfully applied to low resolution images and have been shown less effective for medium/large resolution. 128 × 128 resolution appears to be just in the limit of the capabilities of current DCGANs.

When applying unconditional GANs to image classification tasks, the main idea is to train two or more networks separately, one for each class. Then, once training is completed, the generative part is used to generate random artificial images which are fed to the classifier during training to augment available data for each class. One of the first successful examples of this was [25] where they were able to increase sensitivity and specificity of liver lesion classification from 78.6% and 88.4% with classical data augmentation (DA) methods to 85.7% and 92.4% respectively with additional GANs generated images, on a limited dataset of 64 × 64 computed tomography (CT) images.

In this study, we explore more advanced and recent architectures, such as the StyleGAN family of architectures (StyleGAN [16] and StyleGAN2 [15]). These models have been state-of-the-art in the last year with respect to high realistic and high resolution image generation. However, applications of StyleGAN based models are scare in medical imaging, mainly because this kind of architectures need tens of thousands of examples for training. However, recently an in-built data augmentation mechanism to face data scarcity has been proposed [26,27] which has shown good performance with one order of magnitude less amount of data in FFHQ (human faces), AFHQ Dog (dog faces) and BreCaHAD (breast cancer histopathological images [28]) among others.

To our knowledge, there is no previous work applying StyleGANs to ultrasound images. The only previous example we found in medical imaging was applied to whole-body magnetic resonance imaging (wbMRI) image generation [29] in which DCGAN and StyleGAN family of GANs architectures are compared and StyleGAN showed clear benefits.

## 3. Methods

Firstly, we outline how GANs were adapted to work with fetal brain ultrasound images. Then, we outline how deep learning classifiers were applied with/without artificially generated images.

### 3.1. Stylegan2 Applied to Fetal Ultrasound Images

In this section we describe how we adapted Stylegan2 to fetal brain ultrasound images. We describe the training configuration (Section 3.1.1), the evaluation metrics used for network selection (Section 3.1.2) and the procedure for artificial image generation (Section 3.1.3).

#### 3.1.1. GANs Training

Since we are approaching this problem under an unconditional framework, two GANs were trained, one for trans-thalamic (TTA) and another for trans-ventricular (TRV). Our base architecture is based on Stylegan2-ada [27] where the parameter search space of the network is very broad. Due to time and computing limitations, some parameters were fixed following the author’s recommendations given the dataset size, while the more sensitive were evaluated and set differently for TTA and TRV images. Please see Supplementary Material for a more detailed description of all training parameters used.

#### 3.1.2. GANs Evaluation

It has been proved that GANs are remarkably effective at generating both high-quality and varied artificial images in a broad range of applications. However, GANs lack an objective function, which difficults the comparison between different models. Several quantitative measures have been proposed to evaluate GANs performance. The two more popular metrics for evaluating GANs are Fréchet inception distance (FID [30]) and Precision and Recall (PR [31]).

##### Fréchet Inception Distance

FID is defined in Equation (1), as the Fréchet distance (also known as Wasserstein-2 distance) between two multidimensional Gaussian distributions, $g=(\mu_{g},\Sigma_{g})$ and $r=(\mu_{r},\Sigma_{r})$, representing embedding feature spaces defined by a specific intermediate layer of a pre-trained Inception network of generated and real images respectively.

(1) $$\mathrm{FID}\left(r,g\right)=\left|\right|\mu_{r}-\mu_{g}{\left|\right|}_{2}^{2}+tr\left(\Sigma_{r}+\Sigma_{g}-2{\left(\Sigma_{r}\Sigma_{g}\right)}^{\frac{1}{2}}\right)$$

where *tr* is the trace function of a matrix A

$$tr\left(A\right)=\sum_{i=1}^{n}a_{ii}=a_{11}+a_{22}+\cdots +a_{nn}$$

##### Precision and Recall

The main idea of PR metric is to form explicit non-parametric representations of real and generated manifolds and estimate from them precision and recall. Similarly to FID, real and generated images are embedded into a high-dimensional feature space using a pre-trained classifier network (VGG16). Let $\phi_{r}$ and $\phi_{g}$ be real and generated feature vectors respectively, and $\Phi_{r}$ and $\Phi_{g}$ the corresponding sets of feature vectors. Then for any ϕ and any Φ a binary function is defined as in Equation (2).

(2) $$f\left(\phi ,\Phi \right)=\left\{\begin{array}{cc} 1, & \mathrm{if}\;||\phi -\phi^{\prime }{\left|\right|}_{2}\leq ||\phi -NN_{k}\left(\phi^{\prime },\Phi \right){\left|\right|}_{2}\;\mathrm{for}\;\mathrm{at}\;\mathrm{least}\;\mathrm{one}\;\phi^{\prime }\in \Phi \\ 0, & \mathrm{otherwise} \end{array}\right.$$

where $NN_{k}\left(\phi^{\prime },\Phi \right)$ is the *k*th nearest feature vector of $\phi^{\prime }\in \Phi$.

This equation defines a way to decide whether a given image looks realistic or might be produced by the generator with $f(\phi ,\Phi_{r})$ and $f(\phi ,\Phi_{g})$ respectively.

Finally, precision and recall are defined in Equations (3) and (4)

(3) $$\mathrm{precision}\left(\Phi_{r},\Phi_{g}\right)=\frac{1}{|\Phi_{g}|}\sum_{\phi_{g}\in \Phi_{g}}f\left(\phi_{g},\Phi_{r}\right)$$

(4) $$\mathrm{recall}\left(\Phi_{r},\Phi_{g}\right)=\frac{1}{|\Phi_{r}|}\sum_{\phi_{r}\in \Phi_{r}}f\left(\phi_{r},\Phi_{g}\right)$$

According to prior work, on small datasets, FID is reportedly not a good metric, while precision and recall both have small bias and of the two precision performs better than recall [27,31,32]. With this in mind, we used precision as the main metric for network selection. Despite its shortcomings, we decided to report also FID in our experiments to make easier the comparison with prior studies, which often report it.

#### 3.1.3. Artificial Image Generation

An open problem in all generative models is the difficulty for the generator to learn from low density areas that are poorly represented. In order to improve quality of samples, a method call truncation trick [33] is widely used. The main idea consists in sampling from a truncated distribution instead that from the original distribution (N(0,I) or U[−1,1] in most cases). Sampling from these truncated distributions will generate in most of the models more realistic images, increasing precision at the price of less variety or recall.

This truncation procedure is controlled by a threshold ψ∈(−∞,+∞) and previous works [15,16,27] have shown that values around ψ=0.7 improve the quality of images. As the threshold gets closer to 0 (ψ→0), the generated images tend to be similar to the training average image. On the contrary, when ψ increases, generated images show higher detail but also might present artifacts and seem unrealistic.

The selection of ψ value when generating images is an important parameter to evaluate since it can provide very different results. We performed a grid search optimization on its value to find its optimal value. In the stylegan architecture family, the truncation is done over an intermediate latent space w∈W [27] coming from a mapping network consisting of 8 fully connected layers, instead of traditional latent code y∈Z defined by the input layer. Although in theory negative values of ψ are possible, we didn’t experiment in this work with them, limiting the grid search only to positive values.

### 3.2. Classifiers

The main goal of this work is to study the feasibility of GANs to improve classification. As baseline classifier, we used a Resnet [34] pre-trained on ImageNet dataset, slightly modified by fastai library (see Supplememtary materials on the differences with original). The classifier was trained with and without artificially generated images to compare its results. In all experiments, classifiers were trained a maximum of 20 epochs with early stopping on validation loss with patience set to 5 and batch size to 64. The network was fine tuned the first epoch, by training only the head, while in the remaining epochs, all the layers were unfrozen and trained. All hyper-parameters were set using default fastai values with *fine_tune* method. Moreover, loss function and optimizer were set to default values of *create_lerner* method. Finally, also default data augmentation was applied with method *aug_transforms* with image resolution of 128 × 128. As we mention previously, all these default values define good baselines.

All experiments were done in Google Colab with our own stylegan2-ada (https://github.com/albertoMontero/stylegan2-ada, accessed on 14 January 2021) fork with modifications and custom training configurations.

## 4. Experiments

We first introduce the dataset used in all experiments (Section 4.1). Then, we show GANs results (Section 4.2). Finally, we present and discuss classification results (Section 4.3).

### 4.1. Fetal Brain Ultrasound Images

For all experiments, we used the open-source dataset provided with paper [8] and openly available from zenodo [13]. These images were collected by BCNatal, a center with two sites (Hospital Clinic and Hospital Sant Joan de Deu, Barcelona, Spain), with large maternal-fetal experienced practice. Images were acquired from a total of six different US machines by several different operators with similar experience. The final dataset is composed of 8747 images, 3436 for TRV and 5311 for TTA. Then, all images were cropped by means of an automatic brain detector based on a convolutional neural network, trained on thousands of fetal ultrasound brain images [35]. Figure 1 shows some image examples.

The dataset was partitioned using two different train-validation splits depending on the experiment, always using patient ID to avoid overlapping patient samples, see Table 1. In augmentation experiments, both for GANs and classifiers, 50% of the images were used to train and the remaining 50% were left for validation. In replacement experiments, in order to be fair, we avoided replacing real samples with artificial images generated using those samples during GAN training, and therefore we used 50 + 25% for training and 25% for validation, where half of the previous validation was added to a new training category reserved for the Classifier’s training only.

### 4.2. GAN Training Results

With the configurations outlined in Section 3.1.1 we trained a TTA-GAN for about 45 h and a TRV-GAN for about 27 h in a single GPU. The result of both GANs is shown in Table 2. The obtained values are comparable with numbers previously obtained in [27] for BRECAHAD dataset ([28]) which consists of breast cancer histopathology images with similar number of training images (1994, compared with 1656 for TRV and 2620 for TTA). They obtained a FID value of 15.71 when training from scratch and 16.33 when using transfer learning.

**Figure 2:**
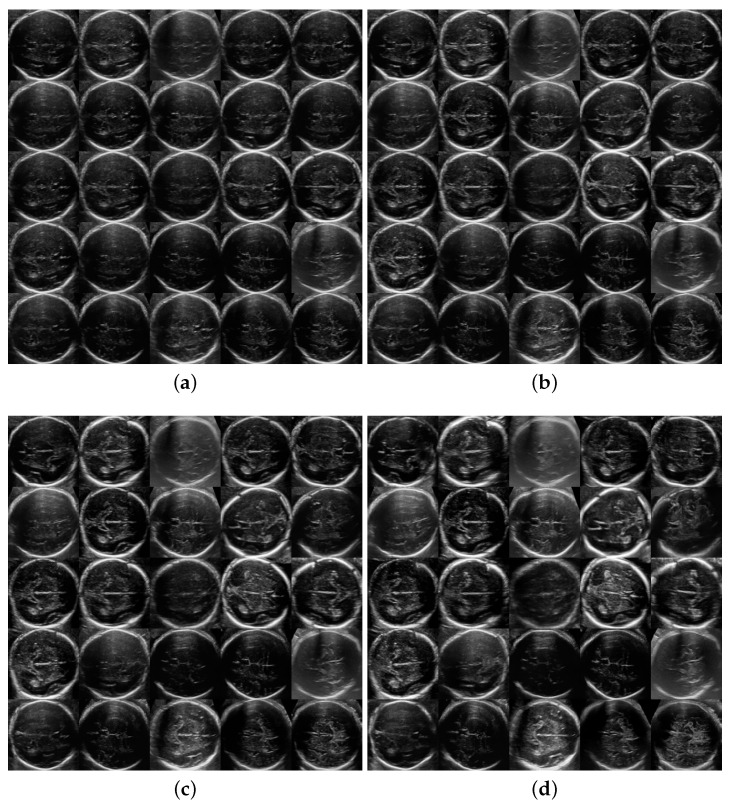
Generation of Trans-thalamic images for some random seeds and different ψ. Same 25 seeds were applied to each grid giving the same 25 brain plane generation for three ψ values and no truncation. (**a**) ψ=0.3; (**b**) ψ=0.5; (**c**) ψ=0.7; (**d**) ψ=1 (no truncation).

**Figure 3:**
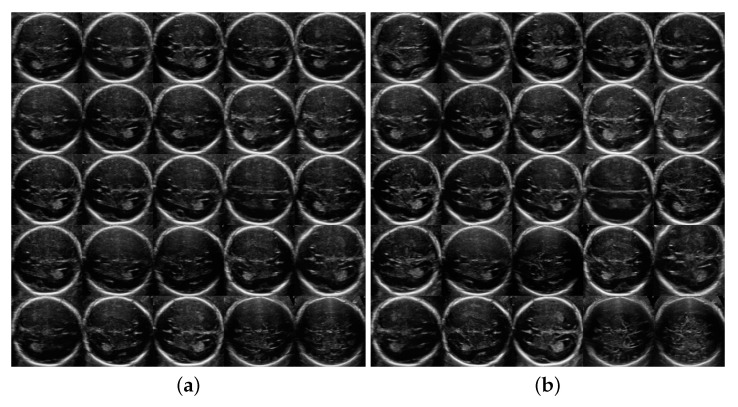

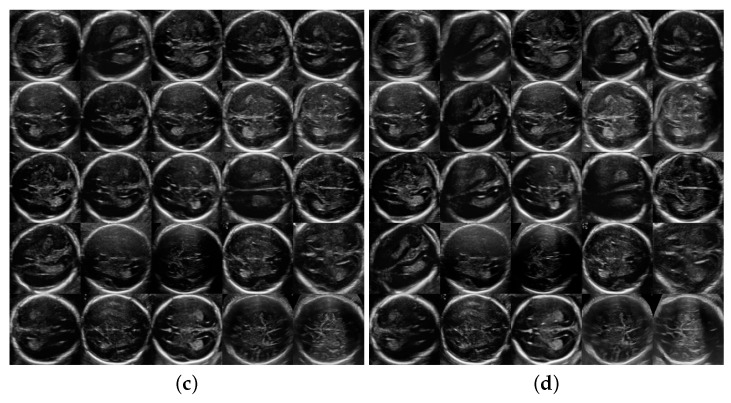
Generation of Trans-ventricular images for some random seeds and different ψ. Same 25 seeds were applied to each grid giving the same 25 brain plane generation for three ψ values and no truncation. (**a**) ψ=0.3; (**b**) ψ=0.5; (**c**) ψ=0.7; (**d**) ψ=1 (no truncation).

Figure 2 and Figure 3 show 25 random artificial generated images for different values of the truncation parameter ψ=0.3,0.5,0.7,1 for both TTA and TRV planes. While low values of ψ generate images very similar between them due to lack of detail, higher values seem to work well, generating realistically looking and varied artificial fetal brain ultrasound images.

### 4.3. Classification of Fetal Brain Ultrasound Images

As explained in Section 3.2, to form a strong classifier, we used a slightly altered ResNet architecture and trained it using latest data augmentation techniques. We tested different depths (Resnet-18 and Resnet-50) and image resolutions to find the best trade-off between performance and training time. Table 3 shows the results. Due to computational resources limitation, and since we aim to perform explorations over ψ truncation parameter with different augmentation ratios and five runs, we decided to perform all experiments using configuration *ResNet18_128x128* which performs slightly worse than *ResNet18_224x224*, but is 50% faster.

Using *ResNet18_128x128* as baseline, we tested if results could be improved through the use of the GANs in two different scenarios: augmentation experiments (Section 4.3.1) where the training examples are augmented with artificial images, and replacement experiments (Section 4.3.2), where some real training samples are replaced by artificial ones.

#### 4.3.1. Augmentation Experiments

**Figure 4:**
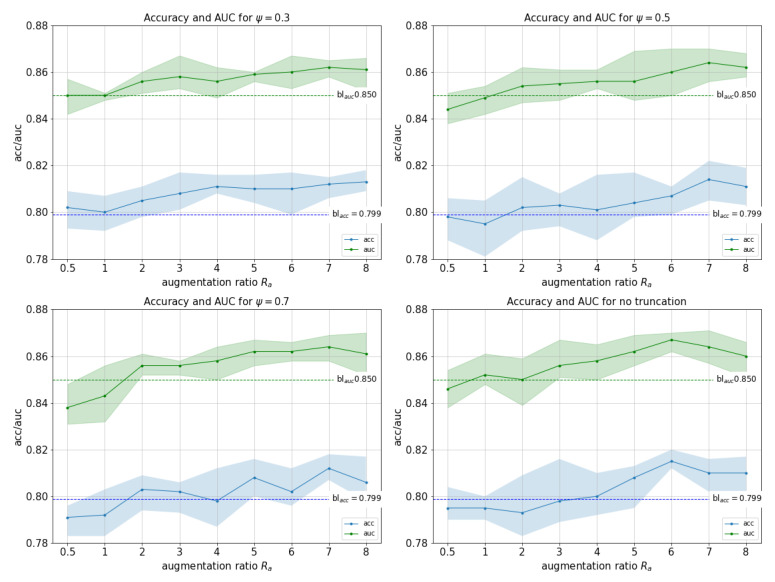
Accuracy (blue, with max and min) and AUC (green, with max and min) for experiments with ψ=0.3, ψ=0.5, ψ=0.7 and ψ=1 (no truncation). Horizontal lines represent the baseline accuracy and AUC (without GAN data augmentation).

The first experiment evaluates whether the performance of the classifier can be improved by augmenting the training set with artificial images generated by the GANs. As we mentioned in the previous section, the quality of images can be controlled by the truncation parameter ψ. We performed several experiments for values ψ=0.3,0.5,0.7,1. We also experimented with the ratio of artificial to real images used during training $R_{a}=\frac{\#\mathrm{artificial}}{\#\mathrm{real}}$, where #real is the total number of real images and is constant. Figure 4 shows graphs for all experiments.

Best results were reached with ψ=1 and $R_{a}=6$ with a maximum accuracy of 81.5% (1.6% improvement over baseline), and a maximum AUC of 86.7% (1.7% improvement over baseline). This shows that even when combined with strong data augmentation methods, GAN-based augmentation can still improve performance. We observed this improvement for all ψ values explored as long as $R_{a}\geq 2$.

When comparing performance for different $R_{a}$ values, performances are very similar to each other. Best $R_{a}$ values are $R_{a}\geq 6$ and at $R_{a}=8$ performance starts to decrease. However, we noticed some differences with respect to parameter ψ. With ψ=0.3, which represents images with higher precision and lower recall, classifiers improve on baseline accuracy and AUC even with low $R_{a}$ values, while the rest need more samples to reach similar results. This means that when adding few fakes ($R_{a}\leq 3$) the quality (precision) matters, but as we add more, variety (recall) seems to compensate quality. As far as overall ψ values are concerned, differences found in terms of best performance were extremely narrow, with fluctuations below 1% in terms of AUC.

To establish whether differences in performance between the best model and the baseline are statistically significant, we performed a permutation test (100 repetitions using stratified k-fold at various K=2,5,10). In all cases, *p*-value for both ACC and AUC was p<0.01, indicating that the improvement is indeed statistically significant.

Please see Supplementary Material for Tables with full results of each one of these experiments.

##### Comparison with Classic Data Augmentation

For completeness, we now compare performance of the best model (ψ=1, $R_{a}=6$) against different baseline scenarios: no data augmentation, classical data augmentation only and GAN-based data augmentation only. Table 4 shows the results. We observe that GANs-based data augmentation on its own improves performance compared with no data augmentation, but does not improve classical data augmentation. However, as previously reported in Figure 4, both techniques are complementary and combining both does improve performance.

We believe that there are two main reasons why classical data augmentation on its own outperformed GAN-based data augmentation:

1. The regular data augmentation used in our paper (*aug_transforms*, from fastai library as mentioned in Section 3.2) is a very strong, state-of-the-art augmentation. It includes many different transformations such as horizontal flips, rotations, brightness and contrast transformations, etc. These transformations and the defaults set by fastai have been found after many experiments and reach strong performance in most scenarios.
2. While in the case of classical data augmentation, all training samples are real images, in GAN-based augmentation many are fake. Generated samples by GANs differ in quality, some being better than others. GANs metrics reported in this work (FID and PR) don’t provide information on the quality of individual samples. A procedure for filtering poor quality images might be worth exploring and potentially give better performance and/or reduce the necessity for so large values of $R_{a}$.

#### 4.3.2. Replacement Experiments

In replacement experiments, instead of augmenting the training set with fakes, we directly replace real images by artificial ones. With this kind of experiment we aim to answer a different question: can GANs help to reach similar performance using less real training images?

**Figure 5:**
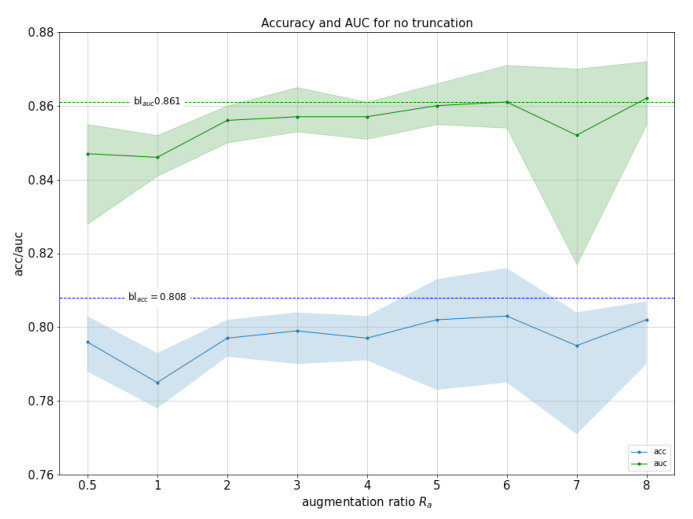
Accuracy (blue, with max and min) and AUC (green, with max and min) for replacement experiments for no truncation. Horizontal lines represent the baseline accuracy and AUC (without GAN data augmentation).

Based on augmentation experiments results and given that performance obtained are similar for all ψ values explored, we performed a single replacement experiment for the best truncation value found (no truncation) and the same augmentation ratios as in augmentation experiments. Figure 5 shows the results.

Results show that AUC obtained are similar to baseline for $R_{a}=5$, $R_{a}=6$, and $R_{a}=8$ although accuracy is slightly lower. This means that similar performance is obtained when replacing 2222 (854+1368) real images by 25,656 (9936 + 15,720), 29,932 (11,592+18,340) and 34,208 (13,248 + 20,960) artificial images for $R_{a}=5$, $R_{a}=6$ and $R_{a}=8$ respectively.

These 2222 images are all from different patients (not used during training). Taking into account that there are in average 3.5 images per patient in the dataset, this means that similar performance is achieved with 570 fewer patients. This, in turn, means that similar results would have been achieved while avoiding 570 physical examinations and all the acquisition, collection, selection and storage of the corresponding images by clinicians. Thereby, this contribution would have saved considerable amounts time, resources and money in the whole process. This is clearly a very strong point in favor of GAN-based data augmentation.

Please see Supplementary Materials for Tables with full numerical results of this experiment.

## 5. Discussion

There is not much previous work related to GANs-based data augmentation for classification tasks in real applications with which to compare our study. In [25] they perform GANs-based synthetic computed tomography images for downstream liver lesion classification task and they were able to increase sensitivity and specificity of liver lesion classification from 78.6% and 88.4% with classical data augmentation methods to 85.7% and 92.4%. However, the dataset used was very limited, containing only 182 examples. Another related work is [32] where they observed an increase in top-1 accuracy between 1% and 3% for a very few classes using ImageNet dataset and BigGAN architecture. Finally, in [36] they performed a comparison among several semi-supervised GAN-based data augmentation methods, but did not observe any improvement over classical data augmentation.

As far as we know, this is the first work using state-of-the-art GANs [27], *stylegan2-ada* architectures for classification tasks on ultrasound imaging. Experiments were performed thoroughly, comparing the advantage of using GANs for classification starting from strong baselines with and without latest classical data augmentation techniques. The results provide insights about quality (precision) and variety (recall) trade-off when generating GAN-based artificial images to improve classification, and show that GANs can be used both to improve classification performance as to reach similar results with fewer real images, translating into considerable savings in time, resources and money for the clinics.

We have to mention here some limitations about this work. First, agreement between two human clinical experts when classifying TRV and TTA is 89.3% and 80% respectively [8], meaning that reported performance is already almost on par with them which is perhaps why only small improvements were observed. Secondly, the work was dedicated to binary classification, and we did not explore other scenarios to check whether the results reported in this study could be extended to multi-class. Finally, fetal brain ultrasound images were pre-processed by means of a brain detector so that images were all centred. We didn’t explored how well these architectures perform on raw ultrasound images.

## 6. Future Work

In this study we used about 4 K training images in a binary classification task. Since in many scenarios it might not be possible to gather this amount of data, a future study could research how well these generative models perform with even less data. It seems sensible to think that these models might be helpful in medium-regime data scenarios, while presumably they will not be useful when very few examples are available (since GANs will not be capable of generating good quality images) or when enough data is available (since gains obtained by the application of GANs will be limited, given the good performance of standard data augmentation techniques as observed in this study).

Another interesting future improvement direction could be to research some sort of quality control over the images generated by the network. A more specific metric (perhaps from a derivation of Equation (2)) could be used to automatically filter poor quality images and increase overall performance.

## Figures and Tables

**Table 1:** Train/validation split with no overlapping patient samples used in augmentation and replacement experiments.

**Augmentation Experiments**				
**Plane**	**Train**		**Validation**	**Total**
TRV	1656		1780	3436
TTA	2620		2691	5311
total	4276		4471	8747
**Replacement Experiments**				
**Plane**	**Train**		**Validation**	**Total**
	**GAN**	**Classifier**		
TRV	1656	854	926	3436
TTA	2620	1368	1323	5311
total	4276	2222	2249	8747

**Table 2:** Metrics: FID, precision and recall for TTA and TRV GANs.

Plane	FID	Precision	Recall
TTA	13.08	0.6616	0.3336
TRV	17.4856	0.6609	0.2850

**Table 3:** Baseline comparison. 5 runs with Tesla T4 gpu and bs = 64.

Model	Accuracy	AUC	F1-Score	Sec/Epoch
ResNet18_128x128	0.799±0.004	0.850±0.003	0.785±0.003	15
ResNet50_128x128	0.806±0.005	0.854±0.004	0.787±0.010	26
ResNet18_224x224	0.805±0.004	0.856±0.001	0.789±0.004	23
ResNet50_224x224	0.816±0.004	0.865±0.004	0.801±0.002	66

**Table 4:** Comparison of baseline classifier without and with different strategies of data augmentation (classical and GAN-based using ψ=1, $R_{a}=6$, *ResNet18_128x128*, 5 runs).

	Accuracy	AUC	F1-Score
no DA	0.739±0.005	0.782±0.005	0.720±0.005
classic DA only (baseline)	0.799±0.004	0.850±0.003	0.785±0.003
GAN-based DA only	0.765±0.008	0.812±0.006	0.746±0.007
classic + GAN-based DA	0.815±0.003	0.867±0.003	0.800±0.004

## Data Availability

All data used in this study is openly available online [13]. The code is available from GITHUB: https://github.com/albertoMontero/stylegan2-ada (accessed on 14 January 2021).

## Supplementary Materials

The following are available at https://www.mdpi.com/article/10.3390/s21237975/s1, Section S1: ResNet18 Fastai Architecture, Section S2: GANs Training, Section S3: Augmentation experiments, Section S4: Replacement Experiments, Figure S1: Comparison between the original ResNet pyTorch implementation and the fastai Resnet architecture used in our study, Table S1: GANs training configuration for Trans-thalamic (TTA) and Trans-ventricular (TRV) images. mb: minibatch size. mbstd: minibatch standard deviation layer at the end of the discriminator. fmaps: the ratio of feature maps used with respect high resolution settings. ema: the exponential moving average of generator weights. map: the mapping network depth. aug:augmentation used TL: transfer learning used, Table S2: Augmentation experiment for ψ=0.3 (5 runs). In left column augmentation ratios with respect training set size are shown. Baseline metrics in first row, Table S3: Augmentation experiment for ψ=0.5 (5 runs). In left column augmentation ratios with respect training set size are shown. Baseline metrics in first row, Table S4: Augmentation experiment for ψ=0.7 (5 runs). In left column augmentation ratios with respect training set size are shown. Baseline metrics in first row, Table S5: Augmentation experiment for no truncation (5 runs). In left column augmentation ratios with respect training set size are shown. Baseline metrics in first row, Table S6: Replacement experiment for no truncation (5 runs). In left column augmentation ratios with respect training set size are shown. Baseline metrics in first row.
