# Supplementary material for: Generative Adversarial Networks to Improve Fetal Brain Fine-Grained Plane Classification
Source: Sensors (Basel). 2021 Nov 29;21(23):7975. doi: 10.3390/s21237975 (PMC8659720; doi:10.3390/s21237975)
Supplement: Supplementary file 1 [file sensors-21-07975-s001.zip › sensors-1440418-supplementary.pdf]

sensors

MDPI

Article

Supplementary Materials

S1. ResNet18 Fastai Architecture

As baseline classifier, we used Resnet [1] with pre-trained weights on ImageNet dataset with the architecture modifications from fastai library [2] . The differences between original Resnet and fastai version are two (see also figure S1):

1. One more *Linear* layer with dimensions (in_features, out_features) = (1024, 512) is added before the last layer modifying the dimension of the last one to (512, numClasses) (numClasses=2 in our case since we perform a binary classification task). Moreover, the next to last layer is followed by *ReLU*, *BatchNorm* and *Dropout* (p=0.5) layers and preceded by *Flatten*, *BatchNorm* and *Dropout* (p=0.25). These modifications reportedly increase of performance in classification tasks.
2. The second modification is located in the link between body and head. While original Resnet has an *AdaptiveAvgPool2d* layer connecting the last feature map with the head, fastai version has *AdaptiveAvgPool2d* and *AdaptiveMaxPool2d* layers concatenated. Again, fastai team have experimentally observed that by concatening both *AdaptiveAvgPool2d* and *AdaptiveMaxPool2d* layers performance increases compared to using a unique *AdaptiveAvgPool2d* layer. Notice that in both cases, input image size can be smaller than (224, 224).

check for updates

Citation: Montero, A.; Bonet-Carne, E.; Burgos-Artizzu, X.P. Generative Adversarial Networks to Improve Fetal Brain Fine-Grained Plane Classification. *Sensors* **2021**, *21*, 7975. https://doi.org/10.3390/s21237975

Academic Editor: Giacomo Oliveri

Received: 13 October 2021
Accepted: 26 November 2021
Published: 29 November 2021

Publisher's Note: MDPI stays neutral with regard to jurisdictional claims in published maps and institutional affiliations.

Copyright: © 2021 by the authors. Licensee MDPI, Basel, Switzerland. This article is an open access article distributed under the terms and conditions of the Creative Commons Attribution (CC BY) license (https://creativecommons.org/licenses/by/4.0/).

```
PyTorch head:

(avgpool): AdaptiveAvgPool2d(output_size=(1, 1))
  (fc): Linear(in_features=512, out_features=1000, bias=True)

fastai head:

(1): Sequential(
    (0): AdaptiveConcatPool2d(
      (ap): AdaptiveAvgPool2d(output_size=1)
      (mp): AdaptiveMaxPool2d(output_size=1)
    )
    (1): Flatten(full=False)
    (2): BatchNorm1d(1024, eps=1e-05, momentum=0.1, affine=True,
      track_running_stats=True)
    (3): Dropout(p=0.25, inplace=False)
    (4): Linear(in_features=1024, out_features=512, bias=False)
    (5): ReLU(inplace=True)
    (6): BatchNorm1d(512, eps=1e-05, momentum=0.1, affine=True,
      track_running_stats=True)
    (7): Dropout(p=0.5, inplace=False)
    (8): Linear(in_features=512, out_features=2, bias=False)
  )
```

Figure S1. Comparison between the original ResNet pyTorch implementation and the fastai Resnet architecture used in our study.

S2. GANs Training

In Table S1 configuration of styleGAN2-ada is shown. More specifically, we used a minibatch size of 32, a standard deviation of 4 for the layer at the end of the discriminator, a 0.5 ratio of feature maps and an exponential moving average of 10. Image resolution was fixed to 128x128. Although there are not pre-trained networks for 128x128 image resolution, higher resolution trained networks can be used for 128x128 resolution for transfer learning. In our case we transferred from CelebAHQ pre-trained on 256x256 (celebahq256) images, which has centered objects (faces) similarly as our dataset, although

from a very different domain. Although other pre-trained models are available we didn't explore other possibilities due to computational resource limitations. Augmentations were set to configuration 'bg' (blit and geometric augmentations) which is shown to perform well on gray images. And finally, the mapping network depth was set to 8.

Then, we used a subset of the data to evaluate separately two of the parameters more sensitive to the type of images: R_1 regularization γ and learning rate. After evaluation, for TTA images we set R_1 regularization γ as 0.16, and learning rate to 0.002. For TRV we got best results for $\gamma = 0.24$ and a decreased learning rate of 0.001.

Table S1: GANs training configuration for Trans-thalamic (TTA) and Trans-ventricular (TRV) images. mb : minibatch size. mbstd: minibatch standard deviation layer at the end of the discriminator. fmaps: the ratio of feature maps used with respect high resolution settings. ema: the exponential moving average of generator weights. map: the mapping network depth. aug:augmentation used TL: transfer learning used.

	**mb**	**mbstd**	**fmaps**	**ema**	**target**	**aug**	**map**	**TL**	**lr**	γ
TTA	32	4	0.5	10	0.5	bg	8	celebahq256	0.002	0.16
TRV	32	4	0.5	10	0.5	bg	8	celebahq256	0.001	0.24

S3. Augmentation experiments

Tables from S2 to S5 show results obtained

Table S2: Augmentation experiment for $\psi = 0.3$ (5 runs). In left column augmentation ratios with respect training set size are shown. Baseline metrics in first row.

R_a	$fakes_{trv}$	$fakes_{dbp}$	acc	max_{acc}	min_{acc}	auc	max_{auc}	min_{auc}	$loss_{avg}$
bl	0	0	0.799 ± 0.004	0.805	0.792	0.850 ± 0.003	0.855	0.844	0.460
0.5	828	1310	0.802 ± 0.006	0.809	0.793	0.85 ± 0.006	0.857	0.842	0.466
1	1656	2620	0.8 ± 0.005	0.807	0.792	0.85 ± 0.001	0.851	0.848	0.466
2	3312	5240	0.805 ± 0.004	0.811	0.798	0.856 ± 0.003	0.86	0.851	0.463
3	4968	7860	0.808 ± 0.006	0.817	0.801	0.858 ± 0.005	0.867	0.853	0.455
4	6624	10480	0.811 ± 0.003	0.816	0.808	0.856 ± 0.005	0.862	0.849	0.457
5	8280	13100	0.81 ± 0.004	0.816	0.804	0.859 ± 0.002	0.86	0.856	0.453
6	9936	15720	0.81 ± 0.006	0.817	0.799	0.86 ± 0.005	0.867	0.853	0.453
7	11592	18340	$\mathbf{0.812} \pm 0.003$	0.815	0.806	$\mathbf{0.862} \pm 0.003$	0.865	0.858	0.456
8	13248	20960	$\mathbf{0.813} \pm 0.003$	0.818	0.809	0.861 ± 0.005	0.866	0.852	**0.447**

Table S3: Augmentation experiment for $\psi = 0.5$ (5 runs). In left column augmentation ratios with respect training set size are shown. Baseline metrics in first row.

R_a	$fakes_{trv}$	$fakes_{dbp}$	acc	max_{acc}	min_{acc}	auc	max_{auc}	min_{auc}	$loss_{avg}$
bl	0	0	0.799 ± 0.004	0.805	0.792	0.850 ± 0.003	0.855	0.844	0.460
0.5	828	1310	0.798 ± 0.007	0.806	0.788	0.844 ± 0.005	0.851	0.838	0.482
1	1656	2620	0.795 ± 0.008	0.805	0.781	0.849 ± 0.004	0.854	0.842	0.481
2	3312	5240	0.802 ± 0.008	0.815	0.792	0.854 ± 0.006	0.862	0.847	0.467
3	4968	7860	0.803 ± 0.005	0.808	0.794	0.855 ± 0.005	0.861	0.848	0.461
4	6624	10480	0.801 ± 0.009	0.816	0.788	0.856 ± 0.003	0.861	0.853	0.464
5	8280	13100	0.804 ± 0.007	0.817	0.798	0.856 ± 0.007	0.869	0.848	0.463
6	9936	15720	0.807 ± 0.004	0.811	0.799	0.86 ± 0.006	0.87	0.85	0.454
7	11592	18340	$\mathbf{0.814} \pm 0.006$	0.822	0.805	$\mathbf{0.864} \pm 0.004$	0.87	0.856	**0.453**
8	13248	20960	0.811 ± 0.006	0.819	0.803	0.862 ± 0.004	0.868	0.858	0.455

Table S4: Augmentation experiment for $\psi = 0.7$ (5 runs). In left column augmentation ratios with respect training set size are shown. Baseline metrics in first row.

R_a	fakes_{trv}	fakes_{dbp}	acc	max_{acc}	min_{acc}	auc	max_{auc}	min_{auc}	loss_{avg}
bl	0	0	0.799 ± 0.004	0.805	0.792	0.850 ± 0.003	0.855	0.844	0.460
0.5	828	1310	0.791 ± 0.004	0.796	0.783	0.838 ± 0.006	0.848	0.831	0.484
1	1656	2620	0.792 ± 0.008	0.803	0.783	0.843 ± 0.009	0.856	0.832	0.492
2	3312	5240	0.803 ± 0.005	0.809	0.794	0.856 ± 0.004	0.861	0.852	0.466
3	4968	7860	0.802 ± 0.005	0.806	0.793	0.856 ± 0.003	0.858	0.852	0.465
4	6624	10480	0.798 ± 0.008	0.812	0.787	0.858 ± 0.005	0.864	0.85	0.462
5	8280	13100	0.808 ± 0.006	0.816	0.8	0.862 ± 0.005	0.867	0.856	0.453
6	9936	15720	0.802 ± 0.006	0.812	0.796	0.862 ± 0.003	0.866	0.858	0.453
7	11592	18340	$\mathbf{0.812} \pm 0.004$	0.818	0.807	$\mathbf{0.864} \pm 0.003$	0.869	0.858	**0.446**
8	13248	20960	0.806 ± 0.008	0.817	0.798	0.861 ± 0.007	0.87	0.852	0.455

Table S5: Augmentation experiment for no truncation (5 runs). In left column augmentation ratios with respect training set size are shown. Baseline metrics in first row.

R_a	fakes_{trv}	fakes_{dbp}	acc	max_{acc}	min_{acc}	auc	max_{auc}	min_{auc}	loss_{avg}
bl	0	0	0.799 ± 0.004	0.805	0.792	0.850 ± 0.003	0.855	0.844	0.460
0.5	828	1310	0.795 ± 0.005	0.804	0.79	0.846 ± 0.006	0.854	0.838	0.47
1	1656	2620	0.795 ± 0.004	0.8	0.79	0.852 ± 0.005	0.861	0.848	0.472
2	3312	5240	0.793 ± 0.009	0.809	0.783	0.85 ± 0.007	0.859	0.839	0.471
3	4968	7860	0.798 ± 0.009	0.816	0.789	0.856 ± 0.005	0.867	0.851	0.465
4	6624	10480	0.8 ± 0.006	0.81	0.792	0.858 ± 0.005	0.865	0.85	0.465
5	8280	13100	0.808 ± 0.007	0.813	0.795	0.862 ± 0.006	0.869	0.856	0.455
6	9936	15720	$\mathbf{0.815} \pm 0.003$	0.82	0.812	$\mathbf{0.867} \pm 0.003$	0.87	0.862	**0.441**
7	11592	18340	0.81 ± 0.006	0.816	0.802	0.864 ± 0.005	0.871	0.857	0.451
8	13248	20960	0.81 ± 0.008	0.817	0.795	0.86 ± 0.006	0.866	0.851	0.449

S4. Replacement Experiments

Table S6: Replacement experiment for no truncation (5 runs). In left column augmentation ratios with respect training set size are shown. Baseline metrics in first row.

R_a	fakes_{trv}	fakes_{dbp}	acc	max_{acc}	min_{acc}	auc	max_{auc}	min_{auc}	loss_{avg}
bl	0	0	$\mathbf{0.808} \pm 0.005$	0.815	0.803	0.861 ± 0.004	0.867	0.854	0.444
0.5	828	1310	0.796 ± 0.006	0.803	0.788	0.847 ± 0.01	0.855	0.828	0.471
1	1656	2620	0.785 ± 0.006	0.793	0.778	0.846 ± 0.005	0.852	0.841	0.475
2	3312	5240	0.797 ± 0.004	0.802	0.792	0.856 ± 0.004	0.86	0.85	0.467
3	4968	7860	0.799 ± 0.005	0.804	0.79	0.857 ± 0.005	0.865	0.853	0.459
4	6624	10480	0.797 ± 0.005	0.803	0.791	0.857 ± 0.004	0.861	0.851	0.467
5	8280	13100	0.802 ± 0.01	0.813	0.783	0.86 ± 0.005	0.866	0.855	0.456
6	9936	15720	0.803 ± 0.011	0.816	0.785	0.861 ± 0.006	0.871	0.854	0.456
7	11592	18340	0.795 ± 0.012	0.804	0.771	0.852 ± 0.018	0.87	0.817	0.468
8	13248	20960	0.802 ± 0.007	0.807	0.79	$\mathbf{0.862} \pm 0.006$	0.872	0.855	0.459

References

1. He, K.; Zhang, X.; Ren, S.; Sun, J. Deep Residual Learning for Image Recognition. *arXiv* **2015**, arXiv:cs.CV/1512.03385.
2. Paszke, A.; Gross, S.; Massa, F.; Lerer, A.; Bradbury, J.; Chanan, G.; Killeen, T.; Lin, Z.; Gimelshein, N.; Antiga, L.; et al. PyTorch: An Imperative Style, High-Performance Deep Learning Library. In *Advances in Neural Information Processing Systems 32*; Wallach, H., Larochelle, H., Beygelzimer, A., d'Alché Buc, F., Fox, E., Garnett, R., Eds.; Curran Associates, Inc.: Red Hook, NY, USA, 2019; pp. 8024–8035.
